# High Ratio of Manure Substitution Enhanced Soil Organic Carbon Storage via Increasing Particulate Organic Carbon and Nutrient Availability

**DOI:** 10.3390/plants14132045

**Published:** 2025-07-03

**Authors:** Xiaoyu Hao, Xingzhu Ma, Lei Sun, Shuangquan Liu, Jinghong Ji, Baoku Zhou, Yue Zhao, Yu Zheng, Enjun Kuang, Yitian Liu, Shicheng Zhao

**Affiliations:** 1 Heilongjiang Academy of Black Soil Conservation and Utilization, Harbin 150086, China; xiaoyuhao1981@sina.com (X.H.); maxingzhu@163.com (X.M.); tufeisuosunlei@163.com (L.S.); shuangquanliu@126.com (S.L.); jinghong_98@163.com (J.J.); zhoubaoku@aliyun.com (B.Z.); zhaoyue2108@163.com (Y.Z.); annadian@163.com (Y.Z.); kuangenjun2002@163.com (E.K.); 2 Key Laboratory of Black Soil Protection and Utilization, Ministry of Agriculture and Rural Affairs, Harbin 150086, China; 3 State Key Laboratory of Efficient Utilization of Arid and Semi-Arid Arable Land in Northern China, Beijing 100081, China; 19853566856@163.com; 4 Key Laboratory of Plant Nutrition and Fertilizer, Ministry of Agriculture and Rural Affairs, Beijing 100081, China; 5 Institute of Agricultural Resources and Regional Planning, Chinese Academy of Agricultural Sciences, Beijing 100081, China

**Keywords:** manure substitution, soil organic carbon storage, soil nutrient stoichiometry, soil enzymatic activity

## Abstract

Replacing partial chemical fertilizers with organic fertilizer can increase organic carbon input, change soil nutrient stoichiometry and microbial metabolism, and then affect soil organic carbon (SOC) storage. A 6-year field experiment was used to explore the mechanism of SOC storage under different ratios of manure substitution in northeast China, with treatments including chemical fertilizer application alone (nitrogen, phosphorus, and potassium, NPK) and replacing 1/4 (1/4M), 2/4 (2/4M), 3/4 (3/4M), and 4/4 (4/4M) of chemical fertilizer N with manure N. Soil nutrients, enzymatic activity, and SOC fractions were analyzed to evaluate the effect of different manure substitution ratios on SOC storage. A high ratio of manure substitution (>1/4) significantly increased soil total N, total P, total K, and available nutrients (NO_3_^−^-N, available P, and available K), and the 4/4M greatly decreased the C/N ratio compared to the NPK. Manure incorporation increased microbial biomass carbon (MBC) by 18.3–53.0%. Treatments with 50%, 75%, and 100% manure substitution (2/4M, 3/4M, and 4/4M) enhanced bacterial necromass carbon (BNC), fungal necromass carbon (FNC), and total microbial necromass carbon (MNC) by 31.9–63.5%, 25.5–107.1%, and 27.4–94.2%, respectively, compared to the NPK treatment. Notably, the increase in FNC was greater than that of BNC as the manure substitution ratio increased. The increasing manure substitution significantly enhanced particulate organic C (POC) and total SOC but did not affect mineral-associated organic C (MAOC). High soil N and P supplies decreased leucine aminopeptidases (LAPs) and alkaline phosphatase activities but increased the activity ratio of β-glucosidase (BG)/(N-acetyl-glucosaminidase (NAG) + LAP). Treatments with 25% manure substitution (1/4M) maintained maize and soybean yield, but with increasing manure rate, the maize yield decreased gradually. Overall, the high ratio of manure substitution enhanced SOC storage via increasing POC and MNC, and decreasing the decomposition potential of manure C and soil C resulting from low N- and P-requiring enzyme activities under high nutrient supplies. This study provides empirical evidence that the rational substitution of chemical fertilizers with manure is an effective measure to improve the availability of nutrients, and its effect on increasing crop yields still needs to be continuously observed, which is still a beneficial choice for enhancing black soil fertility.

## 1. Introduction

The application of chemical fertilizer is a critical practice to enhance soil fertility, whereas chemical fertilizer production consumes a large number of raw materials and energy while leading to serious environmental pollution [1]. China has abundant organic fertilizer resources, which contain huge nutrient reserves and have broad utilization space, and can result in serious environmental pollution if they cannot be properly used [2]. Research found that organic fertilizer can improve soil physical, chemical, and microbial properties, and increased organic C sequestration [3,4]. Generally, manure fertilization is more efficient in enhancing the soil carbon pool than crop residue return [5].

Replacing part of chemical fertilizers with organic fertilizers can lower chemical fertilizer input and improve soil quality, and is an important fertilization measure for achieving sustainable agricultural development [6,7,8]. There are many reports indicating the positive impact of applying organic fertilizers alone or in combination with chemical fertilizers on crop productivity [9,10,11,12]. However, Gong et al. [13] reported that long-term application of organic manure alone or combined with 50% chemical fertilizer nitrogen (N) significantly enhanced soil organic carbon (SOC) and nutrient contents but decreased crop yield by 1.6–25.4% relative to the chemical fertilizer treatment in the North China Plain. Therefore, the appropriate substitution ratio is crucial for achieving high crop yield and soil quality [14].

SOC is divided into different fractions according to its properties—for example, particulate organic C (POC) and mineral-associated organic C (MAOC) according to size and density [15], plant-derived C, and microbial necromass C (MNC) according to C source [16]. After adding organic residue/manure into soils, the high-quality components are easily decomposed and fixed as a part of the soil microbes [17], while the recalcitrant components can form POC by combining soil sand particles [18]. The residue after microbial death is MNC [19], which can combine with soil minerals or occlude within micropores to form MAOC, and small molecular C substrates of organic fertilizer also bind to fine soil particles to form MAOC [20,21].

Soil microbes have different preferences for organic residue utilization. Bacteria preferentially assimilate high-quality fractions such as protein and starch, while fungi primarily assimilate recalcitrant components such as lignin [22]. Because of different C resources, fungal necromass C (FNC) usually presents higher stability than bacterial necromass C (BNC) [23].

The chemical properties of organic fertilizers greatly influence soil nutrient stoichiometry, which further influences microbial growth and metabolic functions and the composition and sequestration of SOC [17]. Crop straw has a high C/N ratio, and its incorporation often leads to a priming effect and increases fungal necromass C, and manure increases more BNC than FNC [24]. Manure (pig manure) application increases MAOC and decreases native SOC decomposition relative to straw amendment and chemical fertilizers treatments [25]. Ma et al. [26] indicated that continuous organic amendments (12 years) significantly increased soil POC and MAOC compared to the chemical fertilizer (N, phosphorus, and potassium, NPK) treatment, and the increment in both POC and MAOC changed as follows: NPK + cattle manure > NPK + green manure > NPK + rice straw. The C/nutrient ratio in organic amendments was the key factor influencing the accumulation of different organic C fractions. Therefore, how different types of manure fertilizer with varied chemical compositions influence SOC fractions and storage needs further clarification.

Soil hydrolytic enzymes are the primary driver of organic material decomposition, and their activities are regulated by soil nutrient limitation or stoichiometry [27]. For example, under the N limitation condition for microbial growth, N-requiring enzyme (N-acetyl-glucosaminidase (NAG) or leucine aminopeptidase (LAP)) activity will be increased to decompose recalcitrant material to seek mineral N [28] and promote soil organic matter (OM) decomposition. However, how different ratios of manure substitution affect SOC composition and storage via influencing soil enzymatic activity and stoichiometry is unclear.

The northeast of China is the main agricultural production area. It is dominated by black soil with a high OM content. Still, recently, soil OM has greatly decreased because of land over-use and few organic fertilizer inputs, which then influences sustainable agriculture development. Increasing organic fertilizer input to enhance soil quality and fertility is necessary for sustaining crop production in this area. A manure substitution experiment was used to study the mechanism of different substitution ratios regulating SOC storage. The objective is to examine the following issues: (i) the effect of different manure substitution ratios on soil fertility, nutrient stoichiometry, and SOC storage, and (ii) the relationship between manure input, soil nutrient stoichiometry and soil enzymatic activity, SOC composition, and storage. In this study, we hypothesized that a high ratio of manure substitution would promote SOC storage by increasing nutrients and recalcitrant C input and decreasing the activity of N- and P-requiring enzymes.

## 2. Results

### 2.1. Soil Fertility

Compared with the NPK treatment, the 1/4M did not change soil pH or nutrient contents, but the 2/4M, 3/4M, and 4/4M increased soil pH, TN, NO_3_^−^-N, TP, AVP, AVK, and DOC by 1.4–11.1%, 12.8–33.1%, 45.7–101.7%, 14.7–26.7%, 11.3–106.5%, 4.2–25.2%, and 41.6–233.8%, respectively. The increment increased with increasing manure substitution ratio, but different treatments did not significantly influence soil NH_4_^+^-N (Table 1).

**Figure 1:**
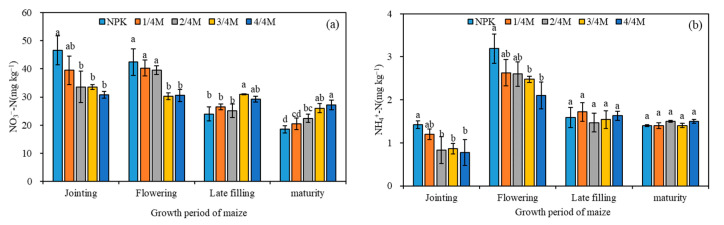
Soil NO_3_^−^-N (**a**) and NH_4_^+^-N (**b**) content in different growth stages of maize. The different lowercase letters in the same period indicate a significant difference at the 0.05 level.

At the jointing and flowering stages of maize, soil NO_3_^−^-N and NH_4_^+^-N in the NPK treatment were significantly higher than those in manure substitution treatments (Figure 1). With the extension of the growth period, soil NO_3_^−^-N and NH_4_^+^-N in the NPK treatment tended to decrease in the late filling stage, and soil NO_3_^−^-N in the mature stage was significantly lower than in the organic substitution treatment.

### 2.2. Soil Organic Carbon Fraction

**Figure 2:**
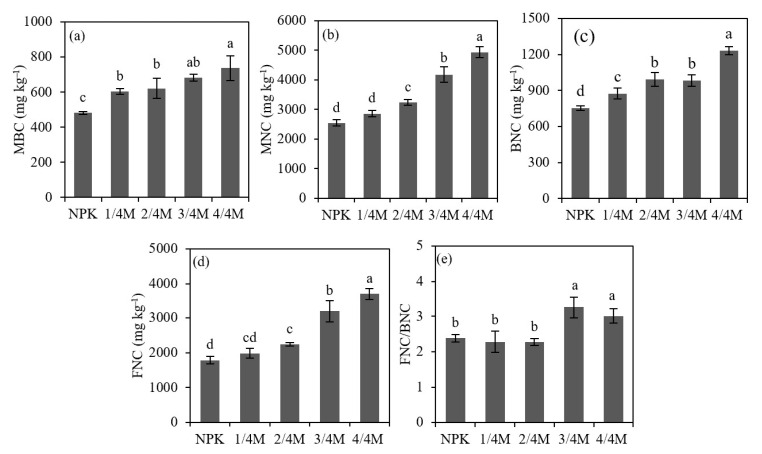
Soil microbial biomass carbon (**a**), microbial necromass carbon (**b**), bacterial necromass carbon (**c**), fungal necromass carbon (**d**) and FNC/BNC ratio (**e**) in different treatments. Note: MBC, microbial biomass carbon; MNC, microbial necromass carbon; BNC, bacterial necromass carbon; FNC, fungal necromass carbon. Different lowercase letters indicate a significant difference at the 0.05 level.

**Figure 3:**
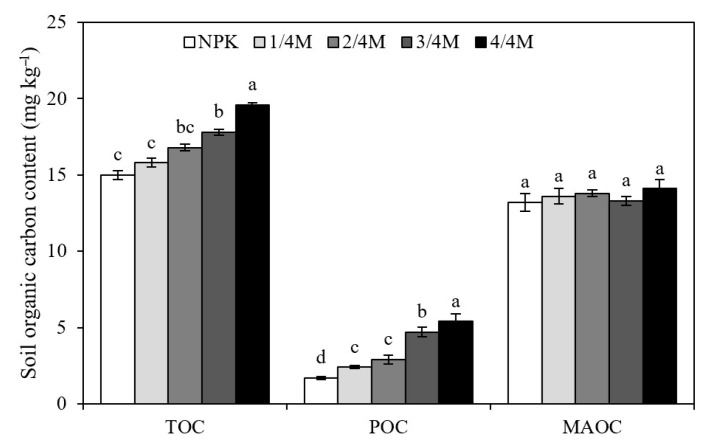
Soil total organic carbon (TOC), particulate organic C (POC), and mineral-associated organic C (MAOC) in different treatments. Different lowercase letters indicate a significant difference at the 0.05 level.

Compared with the NPK treatment, manure substitution treatments increased microbial biomass C by 18.4–53.0%; the 1/4M did not affect soil MNC, FNC, or the FNC/BNC ratio but did significantly increase BNC (Figure 2). The 2/4M, 3/4M, and 4/4M increased the soil BNC, FNC, MNC, and FNC/BNC ratio by 31.9–63.5%, 25.5–107.1%, 27.4–94.2%, and −4.6–37.0%, respectively. Compared with the NPK treatment, the 1/4M, 2/4M, 3/4M, and 4/4M increased POC and TOC by 83.3–233.8% and 12.2–27.5%, respectively, and these organic C fractions generally increased with increasing manure substitution ratio. However, different treatments had no significant effects on MAOC (Figure 3).

### 2.3. Soil Organic Carbon and Nutrient Stoichiometry

**Figure 4:**
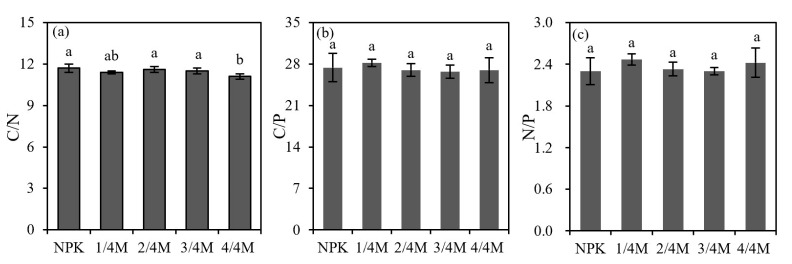
Soil C/N (**a**), C/P (**b**), and N/P ratio (**c**) in different treatments. Different lowercase letters indicate a significant difference at the 0.05 level.

There was no difference in soil C/N ratio among the NPK, 1/4M, 2/4M, or 3/4M treatments, but the 4/4M presented a great decrease relative to the NPK, and the ratios of C/P and N/P were similar across all treatments (Figure 4).

### 2.4. Soil Enzyme Activity and Enzymatic Stoichiometry

**Figure 5:**
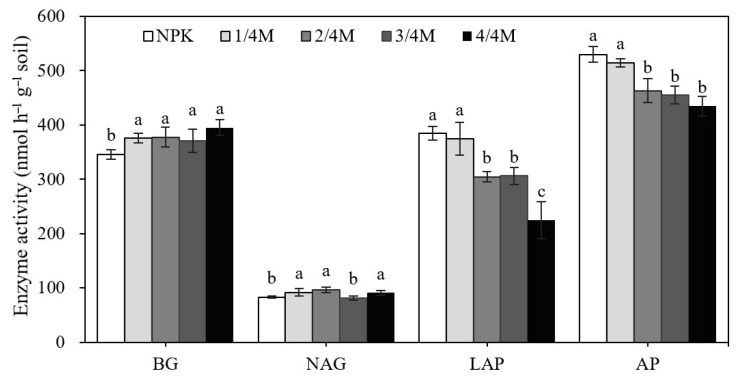
Soil hydrolytic enzymatic activity in different treatments. Note: BG, β-glucosidase; NAG, N-acetyl-glucosaminidase; LAP, leucine aminopeptidase; AP, alkaline phosphatase. Different lowercase letters indicate a significant difference at the 0.05 level.

**Figure 6:**
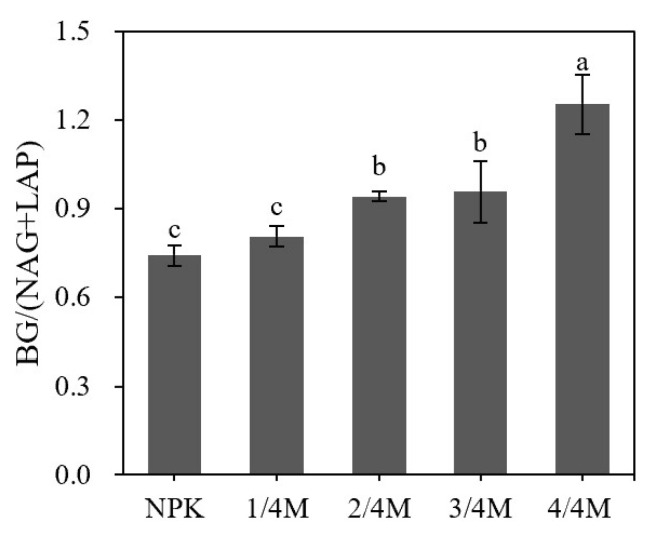
Soil enzyme stoichiometry in different treatments. Note: BG, β-glucosidase; NAG, N-acetyl-glucosaminidase; LAP, leucine aminopeptidase. Different lowercase letters indicate a significant difference at the 0.05 level.

The application of manure enhanced the activity of BG and NAG relative to the NPK treatment, but different manure substitution ratios did not significantly influence their activity (Figure 5). The activity of LAP and AP was similar between the NPK and 1/4M treatments, whereas their activity gradually decreased with increasing manure substitution ratio. The activity ratio of BG/(NAG + LAP) was similar between the NPK and 1/4M, and then the ratio significantly increased with increasing manure substitution ratio (Figure 6).

### 2.5. Partial Least Squares Path Modeling Analyses

**Figure 7:**
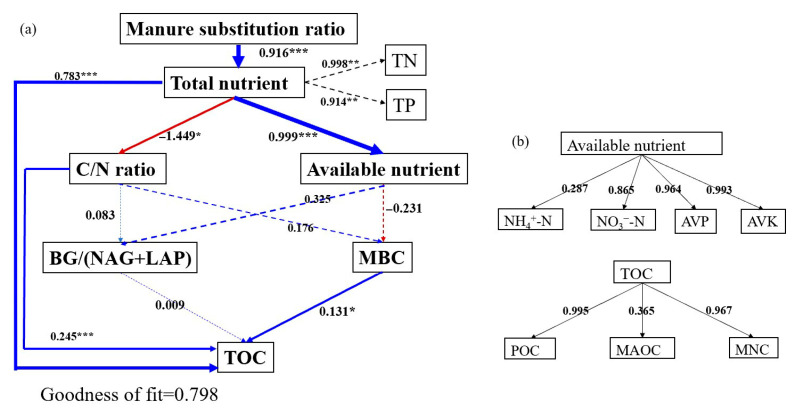
(**a**,**b**) Partial least squares path modeling (PLS-PM) disentangles major pathways of the influences of manure substitution ratio, soil total nutrients (TN, TP), available nutrients (DOC, NO_3_^−^-N, NH_4_^+^-N, available P (AVP), and available K (AVK)), C/N, the activity ratio of soil hydrolytic enzymes, and soil microbes on soil organic carbon storage. Note: Blue and red arrows indicate positive and negative flows of causality (*p* < 0.05), respectively, and dashed lines indicate no significant effect (*p* > 0.05). The path coefficients are calculated after 1000 bootstraps and are reflected in the width of the arrow. The number of asterisks to the upper right of the number indicates the degree of influence: * *p* < 0.05, ** *p* < 0.01.*** *p* < 0.001.

The PLS-PM model was constructed to clarify the cascading relationships among manure substitution ratio, soil nutrient content and stoichiometry, enzyme activity, microbial biomass C, and TOC (Figure 7). This model indicated that different manure substitution ratios had significant, direct positive effects on soil total nutrients (0.916), and soil total nutrients had a significantly positive affect on available nutrients (0.999) and TOC (0.783) and had a negative effect on soil C/N ratio (−1.449). Soil C/N ratio (0.245) and MBC (0.131) showed significantly positive effects on soil TOC. The correction between POC and TOC and between MNC and TOC were greater than that between MAOC and TOC.

### 2.6. Annual Variation in Crop Yield

**Figure 8:**
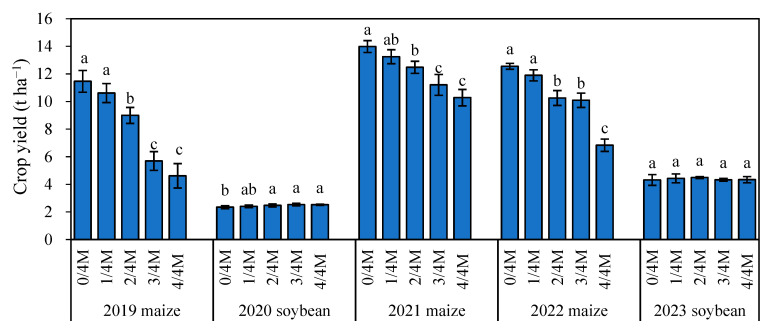
Crop yield in different treatments. Different lowercase letters indicate a significant difference at the 0.05 level.

In each maize season, maize yield did not differ between the NPK and 1/4M treatments but significantly decreased by 21.8–59.8%, 10.9–26.5%, and 18.6–45.7% in 2019, 2021, and 2022, respectively, with increasing manure rate, and the lowest grain yield was in the 4/4M treatment (Figure 8). In soybean season, organic treatment soybean grain yield did not differ among different organic substitution treatments.

## 3. Discussion

### 3.1. Effect of Different Manure Substitution Treatments on Soil Fertility

Replacing chemical fertilizer N with manure N significantly increased soil fertility after the substitution ratio exceeded 25%, because the manure incorporation increased the input of organic C compared to the NPK treatment, and the mineralization and release of manure N and P were slower relative to the chemical fertilizer, thus decreasing the active N and P loss from manure [26]. Similar results have been reported regarding manure application enhancing soil fertility compared to chemical fertilization and crop straw return [25,26]. The NH_4_^+^-N did not differ among fertilization treatments because NH_4_^+^-N derived from chemical fertilizer or manure fertilizer can rapidly convert to NO_3_^−^-N through nitrification under good soil aeration conditions, with little accumulation in soils [29]. Soil pH increased with the increasing manure substitution ratio in this study, which is not consistent with many other studies, in which manure fertilization decreased soil pH [13,25], which may have been the result of different manure fertilizer resources with varied pH, while the horse manure used in this study had a higher pH than local soil (Table 1). These results indicate that the combined application of horse manure with chemical fertilizer could alleviate soil acidification and enhance soil fertility.

### 3.2. Effect of Different Manure Substitution Treatments on Soil Organic Carbon Content and Fractions

The substitution of chemical fertilizer N with manure N significantly increased soil MBC, MNC, POC, and TOC, and these SOC fractions gradually increased with increasing manure substitution ratio. The organic C, N, and P from manure fertilization are important energy and nutrients sources for microbial growth, and can improve MBC accumulation. Microbe-derived C existed in the form of necromass C after microbial death, and the combined application of manure with chemical fertilizer significantly enhanced FNC and BNC relative to chemical fertilization alone [5,25]. In general, MAOC dominated SOC storage in soils. In this study, MAOC did not significantly change among different fertilization treatments, and POC significantly increased with the increasing manure substitution ratio. A similar result was reported by Guo et al. [30] in that the long-term application of manure alone and combined with chemical fertilizer did not affect MAOC but significantly enhanced POC accumulation compared with the NPK treatment in potato farmland. The reason is that manure fertilization enhanced the number of fungi, and soil clay particles formed aggregates by combining with fungal hyphae, leading to the transfer of organic C from MAOC to POC [31]. The content and increment were higher in FNC than in BNC in this experiment. In addition, horse manure has a high content of plant-derived recalcitrant organic C because horses are herbivores, and recalcitrant C fractions easily combine with soil sand particles to form POC. Li et al. [32] also indicated that the proportions of lignin phenols in the SOC and the ratio of lignin phenols to amino sugars in the manure treatments were larger than in the NPK treatment and increased with increasing manure application rate. Meanwhile, continuous manure application may lead to MAOC saturation because of the limitation of the adsorption capacity of minerals for MNC and small molecular C substrates [33].

MNC and POC gradually increased with increasing manure substitution ratio, with POC increasing at a faster rate than MNC. MAOC is formed by combining MNC and soil clay and silt particles, while POC is formed by combining MNC and sand particles, indicating that more MNCs may participate in the formation of large particle aggregates and increase POC content. However, the detailed contribution of MNC to the increase in POC would be confirmed by analyzing the MNC content in POC and MAOC fractions.

### 3.3. Effect of Different Rates of Manure Substitution on Soil Nutrient Stoichiometry and Enzyme Activity

The ratio of C/P and N/P did not differ among fertilization treatments, whereas the C/N ratio decreased significantly in the 4/4M relative to that in the NPK and other manure substitution treatments. The nutrient stoichiometry influences soil microbial growth and function. To meet the demand for nutrients, soil microorganisms will degrade organic matter by secreting hydrolytic enzymes and then regulate SOC storage [34]. For microbial growth, the optimal soil C/N ratio was about 25 [35]. The C/N of the horse manure used (about 16) and that of the original soil (about 10) in this study was lower than 25, so the microorganisms did not need to decompose manure or native organic matter to obtain N. Therefore, the activity of N- and P-acquiring enzymes (LAP and AP) decreased with increasing manure substitution ratio, and the C-acquiring enzyme (BG) did not significantly increase with increasing manure substitution ratio, although manure incorporation enhanced BG activity compared to the chemical fertilization treatment, and the ratio of BG/(NAG + LAP) increased with increasing manure substitution ratio. Therefore, soil total nutrients and the C/N ratio had a significantly positive and direct effect on TOC, and the BG/(NAG + LAP) also presented a positive effect on TOC, although the effect was not significant (Figure 6). In general, high soil microbial activity can promote organic material decomposition because of the demands for organic C; however, soil TOC presented an increased change with increasing manure substitution ratio, so the organic C input from manure or MNC may have been higher relative to the organic C loss resulting from microbial decomposition. Meanwhile, DOC is a labile organic C fraction and is easily utilized by soil microbes; the high amount of DOC supply in the high ratio of manure substitution treatments can alleviate microbial decomposition for stable organic C, protect POC storage, and then promote SOC accumulation. Li et al. [25] reported that NPK + manure enhanced SOC storage compared to NPK alone by strongly decreasing the prime effect, mainly due to the increased N availability. Ma et al. [26] also found that manure application decreased the microbial metabolic quotient and increased microbial C use efficiency and then enhanced SOC storage relative to chemical fertilization treatment.

### 3.4. Effect of Different Rates of Manure Substitution on Crop Yield

Many studies have confirmed that the combined application of organic and inorganic fertilizers can greatly increase crop yields [7,36,37]. The main reason is that the combined application of organic and inorganic fertilizers can improve the soil nitrogen supply process and make the soil nutrients release steadily [38,39]. However, when the organic substitution ratio exceeds a certain proportion, it will affect crop growth and lead to yield reduction. Sun et al. [40] discovered that a 25% manure substitution rate could ensure sustained and considerable yield, but when the manure substitution rate exceeded 50%, yam yield decreased significantly. Shu et al. [41] reported that when the substitution rate exceeds 75%, manure may have a neutral or even negative effect on plant growth. A global meta-analysis report indicated that 100% organic fertilizer substitution would reduce crop yields [42]. In this study, NPK treatment maintained high crop yields, while the organic substitution treatments (especially those with higher substitution rates) decreased maize and soybean yields, aligning with the findings of Li et al. [43] and Yang et al. [44]—that is, the effect of organic substitution treatment on yield increase was not obvious in the first 10 years of the experiment. Because the mineral N release from manure was low, the nutrient demand of the crop growth period could not be fully met, which was proven by the soil NO_3_^−^-N content in different growth stages (Figure 1), thus affecting early crop growth and resulting in late reproductive growth being affected, ultimately reducing crop yield [44,45]. It has been reported that the cumulative absorption of N in the maize seedling stage, vegetative growth stage (from seedling stage to silking), and reproductive growth stage (from silking to maturity) is 2.5%, 85%, and 12.5%, respectively [46]. Therefore, it is particularly important to ensure sufficient nitrogen supply in the vegetative growth stage of maize. Under the conditions of this experiment, the changes in crop yield were further observed and evaluated with the increase in soil fertility and nutrient accumulation caused by continuous application of organic fertilizer. It needs to be affirmed that from the perspective of maintaining sustainable agricultural development, balanced chemical fertilizer combined with organic fertilizer may be the best fertilization method for improving crop yield and soil fertility.

## 4. Materials and Methods

### 4.1. Experimental Site Description

The study was conducted in Minzhu experimental station, Harbin city, Heilongjiang Province, China (126°51′ E, 45°50′ N and altitude 151 m), in 2017. This region has a temperate continental monsoon climate; the average annual temperature and mean annual precipitation are 3.5 °C and 533 mm, respectively; and 60–80% of the precipitation occurs in summer (July to September). The soil in this site is a sandy loam black soil (Haplic Phaeozem, FAO, Rome, Italy) and contains 46.1% sand, 23.8% silt, and 30.1% clay. The initial soil characteristics of the 0–20 cm layer are listed as follows: pH 7.1 (*w*/*v*, 1:2.5), organic matter 31.2 g kg^−1^, total N (TN) 1.9 g kg^−1^, alkali-hydrolyzable N 199.1 mg kg^−1^, available P 41.1 mg kg^−1^, and exchangeable K 215.0 mg kg^−1^.

### 4.2. Experimental Design

This study included five treatments with four replications: (1) N, P, and K nutrients, all of which came from chemical fertilizer (NPK); (2) 3/4 chemical fertilizer N and 1/4 manure N (1/4M); (3) 2/4 chemical fertilizer N and 2/4 manure N (2/4M); (4) 1/4 chemical fertilizer N and 3/4 manure N (3/4M); and (5) 4/4 manure N (4/4M). All treatments were arranged in a randomized complete block design, and the plot area was 4 m^2^ (2 m × 2 m).

Only one season crop is planted every year in this area, and a maize–maize–soybean rotation system was used in this study; maize or soybean was planted in the earth in May and was harvested in late September. In the NPK treatment, the N, P, and K fertilizers were applied at 150 kg N ha^−1^, 75 kg P_2_O_5_ ha^−1^, and 75 kg K_2_O ha^−1^ in the maize season and at 45 kg N ha^−1^, 60 kg P_2_O_5_ ha^−1^, and 45 kg K_2_O ha^−1^ in the soybean season, respectively. Total N, P_2_O_5_, and K_2_O rate was the same in all treatments. The fertilizers used were urea (N 46%), calcium superphosphate (P_2_O_5_ 46%, CaO 20%), and potassium chloride (K_2_O 60%, Cl^−^ 45%). The manure of the organic substitution treatment was decomposed horse manure. The physicochemical properties based on the drying matter basis were as follows: moisture content 70%, pH 9.27, OM 41.8%, TN 1.52%, P_2_O_5_ 1.85%, K_2_O 0.49%, and C/N ratio 16.0. The total N, P_2_O_5_, and K_2_O rate was same in all treatments. The manure application rates of 25%, 50%, 75%, and 100% manure substitution treatments were 8.2, 16.4, 24.7, and 32.9 t ha^−1^, respectively.

In the maize season, 50% of N fertilizer and all P and K fertilizers were applied as basal fertilizer before tillage, and 50% of N fertilizer was band-applied as a top-dressing fertilizer at the 6-leaf stage. In the soybean season, all fertilizers were applied as a basal fertilizer. After the last crop was harvested in late September, all the manure fertilizers were evenly applied to the plot and artificially mixed into the 20 cm soil layer as a basal fertilizer.

### 4.3. Crop Harvest and Soil Sample Collection

At maturity each year, all crops were harvested in every plot, air-dried grains were weighed, and grain yield was reported at the standard moisture of 14.0%. In September 2023, three surface (0–20 cm) soil cores (3 cm in diameter) were collected from each plot after soybean harvest. Three fresh soil samples per plot were mixed and sieved to pass a 2 mm mesh, and then were carried to the laboratory in an icebox immediately. The NH_4_^+^-N, NO_3_^−^-N, microbial biomass C (MBC), and hydrolytic enzyme activity were immediately determined using fresh soil samples, and the remaining subsamples were air-dried for other mineral nutrient and organic C fraction analysis. In 2024, soil samples were collected at the jointing stage, flowering stage, late grain filling stage, and mature stage of corn using the same method as mentioned above to determine NO_3_^−^-N and NH_4_^+^-N.

### 4.4. Soil Physicochemical Property Analysis

Soil NO_3_^−^-N and NH_4_^+^-N were extracted with 1 mol L^−1^ KCl and analyzed using a flow injection analyzer (AA3, SEAL Company, Rottenbach, Germany); TN, TP, AVP, and AVK were determined according to Lu [47]. Dissolved organic C (DOC) was extracted and determined according to the methods described by Cheng et al. [48].

### 4.5. Separation and Determination of Soil Organic Carbon Fractions

Soil POC and MAOC fractions were separated based on particle size using the method of Sokol et al. [49]. Twenty grams of air-dried and sieved (2 mm) soil was placed in a 100 mL centrifuge tube and mixed with 60 mL of sodium hexametaphosphate solution. The mixture was shaken for 18 hours and separated into the POC (>53 μm) and MAOC fractions (<53 μm) using a 53 μm sieve, with the precipitation after centrifugation of the suspension also classified as MAOC [50]. All components were dried at 65 °C, weighed, and then ground with a ball mill and analyzed for organic C using an elemental analyzer (Elementar Analysensystem GmbH, Langenselbold, Germany).

### 4.6. Soil Biological Property Analysis

Soil microbial biomass carbon (MBC) was determined using the chloroform fumigation–extraction method [51]. The activity of hydrolytic enzymes—including β-glucosidase (BG), N-acetyl-glucosaminidase (NAG), leucine aminopeptidase (LAP), and alkaline phosphatase (AP)—was measured using a microplate fluorometric assay with 96-well black microplates and methylumbelliferone (MUB)-linked substrates [52,53].

### 4.7. Amino Sugar Analysis

Amino sugars were extracted and measured according to the procedure described by Zhang and Amelung [54]. Briefly, the air-dried and sieved (0.25 mm) soil samples were mixed with 10 mL HCl (6 mol L^−1^) and hydrolyzed for 8 h at 105 °C, and 100 μL myoinositol was added as an internal standard. The solution was filtered, adjusted to pH 6.6–6.8, and centrifuged at 3000 rpm for 10 min. The hydrolysate was freeze-dried, and the residue was washed with methanol. The recovered amino sugars were transformed into malononitrile derivatives by adding the derivatization reagent acetic anhydride and measured using an Agilent 6890A GC (Agilent, Santa Clara, CA, USA) equipped with a FID and an HP-5 capillary column. The methylglucamine was added to the solution before derivatization to monitor the recovery efficiency of the amino sugars. Glucosamine (GluN) and muramic acid (MurN) were selected to estimate the fungal and bacterial necromass [55]. The FNC was determined based on the difference between total GluN and bacterial GluN combined with the assumed ratio of MurN to GluN (1:2) in bacterial cells. The FNC and BNC (mg C g^−1^ soil) were calculated based on Equations (1) and (2) [19].

FNC = (GluN/179.2 − 2 × MurN/251.2) × 179.17 × 9(1)

BNC = MurN × 45(2)

where 179.2 and 251.2 are the molecular weights of GluN and MurN, and 9 and 45 are the conversion factors of fungal GluN to FNC and bacterial MurN to BNC, respectively. MNC was estimated by the sum of FNC and BNC.

### 4.8. Statistical Analyses

All data were analyzed using SPSS 20.0 for Windows (SPSS, Inc., Chicago, IL, USA), and the treatment means were compared using one-way ANOVA followed by the least significant difference (*p* < 0.05). We analyzed the relationship between the manure substitution ratio, soil nutrient content and stoichiometry, enzyme activity, and SOC fractions using a partial least squares path model (PLS-PM) [56], and the path coefficients and determination coefficients (R2) were estimated with the software package “plspm” (1000 bootstraps) in R (4.4.1).

## 5. Conclusions

In this study, replacing chemical fertilizer with a high ratio of manure fertilizer (>1/4) significantly enhanced soil fertility relative to chemical fertilizer alone. The high nutrient supply promoted microbial growth and MNC accumulation, and decreased the potential decomposition for organic C via decreasing the activity ratio of BG/(NAG + LAP). In addition, manure incorporation increased the input of recalcitrant C fraction and promoted SOC storage. Treatments with 25% manure substitution (1/4M) maintained maize and soybean yield, but with the increase in manure substitution, the maize yield decreased gradually. Therefore, the substitution of partial chemical fertilizer with manure is an efficient measure to increase soil fertility and SOC storage, but the effect of not increasing crop yield in the short term needs to be further verified and evaluated. Future research should explore the applicability of different manure substitution ratio strategies in crop cultivation in black soil regions, especially focusing on the synergistic effect of soil fertility and crop yield to optimize region-specific agricultural policies.

## Figures and Tables

**Table 1:** Soil chemical properties and nutrient contents in different treatments.

Treatment	pH	TN (g kg^−1^)	NH_4_^+^-N (mg kg^−1^)	NO_3_^−^-N (mg kg^−1^)	TP (g kg^−1^)	TK (g kg^−1^)	AVP (mg kg^−1^)	AVK (mg kg^−1^)	DOC (mg kg^−1^)
NPK	6.36 ± 0.18 c	1.3 ± 0.01 c	1.4 ± 0.02 a	18.5 ± 1.3 d	0.56 ± 0.03 c	23.2 ± 0.1 c	29.2 ± 1.4 cd	190.3 ± 5.7 c	7.4 ± 0.2 c
1/4M	6.62 ± 0.1 c	1.4 ± 0.04 c	1.4 ± 0.07 a	20.4 ± 2.1 cd	0.56 ± 0.02 c	23.6 ± 0.2 c	28.2 ± 1.3 d	198.2 ± 5.7 bc	8.1 ± 0.6 c
2/4M	6.93 ± 0.14 b	1.5 ± 0.04 bc	1.5 ± 0.02 a	22.4 ± 1.5 bc	0.62 ± 0.01 b	24.2 ± 0.1 bc	32.8 ± 2.4 bc	199.1 ± 9.2 bc	9.8 ± 0.7 bc
3/4M	7.05 ± 0.27 ab	1.5 ± 0.05 b	1.4 ± 0.05 a	26.0 ± 1.6 ab	0.64 ± 0.02 b	25.6 ± 0.2 ab	36.1 ± 1.7 b	206.7 ± 6.6 b	10.4 ± 1.1 b
4/4M	7.29 ± 0.1 a	1.7 ± 0.07 a	1.5 ± 0.04 a	27.2 ± 1.7 a	0.71 ± 0.05 a	27.4 ± 0.2 a	60.3 ± 1.8 a	238.9 ± 5.6 a	13.6 ± 1.3 a

Note: TN, total nitrogen; TP, total phosphorus; TK, total potassium; AVP, available phosphorus; AVK, available potassium; DOC, dissolved organic carbon. Different lowercase letters in the same column indicate a significant difference at the 0.05 level.

## Data Availability

The original contributions presented in the study are included in the article; further inquiries can be directed to the corresponding author.
